# Comparison of analgesic efficacy, anti-inflammatory effect, and myotoxicity of ultrasound-guided suprainguinal fascia iliaca block and adductor canal with IPACK combination in patients undergoing total knee arthroplasty under spinal anesthesia: A prospective observational study

**DOI:** 10.1097/MD.0000000000043719

**Published:** 2025-08-08

**Authors:** Şenay Canikli Adigüzel, Caner Genç, Ebru Kayikçi, Ahmet S. Genç, Serhat Durusoy, Adem Yildiz, Serkan Tulgar

**Affiliations:** a Department of Anaesthesiology and Reanimation, Samsun University Samsun Training and Research Hospital, Samsun, Turkiye; b Department of Orthopaedics and Traumatology, Samsun University Samsun Training and Research Hospital, Samsun, Turkiye; c Department of Anesthesiology and Reanimation, Faculty of Medicine, Bahçeşehir University, Göztepe Medical Park Hospital, Istanbul, Turkiye.

**Keywords:** adductor canal block, numerical pain scores, posterior knee capsule local anesthetic injection, suprainguinal fascia iliaca block, total knee arthroplasty

## Abstract

Total knee arthroplasty (TKA) is associated with significant postoperative pain, managed with multimodal analgesia, including regional anesthesia techniques like peripheral nerve blocks. The knee joint’s innervation by both sacral and lumbar plexuses often necessitates combined blocks for effective analgesia. This study aimed to compare the effects of suprainguinal fascia iliaca block (SIFIB) and a combination of popliteal artery and posterior knee capsule injection (IPACK) with adductor canal block (ACB) on 24-hour postoperative pain scores, as well as their impact on inflammatory markers and biochemical indicators of myotoxicity. The study included patients undergoing elective unilateral primary knee arthroplasty. They were divided into 2 groups: 1 received postoperative SIFIB, and the other underwent IPACK preoperatively and ACB postoperatively. Postoperative evaluations included Numeric Rating Scale scores, morphine consumption, quadriceps muscle strength, neutrophil/lymphocyte ratio (NLR), platelet/lymphocyte ratio (PLR), systemic immune inflammation score, C-reactive protein (CRP), lactate, and creatine phosphokinase (CPK) levels as a marker of myotoxicity. CPK increases of more than 5-fold were assessed for rhabdomyolysis. Pain scores at rest and with movement were comparable between groups at all time points (*P* > .05). Morphine consumption over 24 hours did not differ significantly (*P* > .05). Similarly, inflammatory markers, including NLR, PLR, systemic immune inflammation score, CRP, and lactate, showed no significant differences between groups at 12 and 24 hours (*P* > .05). CPK levels, evaluated as indicators of myotoxicity and rhabdomyolysis, were also similar (*P* > .05). Quadriceps strength, assessed as an indicator of motor loss, showed no significant differences between groups (*P* > .05). The analgesic efficacy, safety, and inflammatory responses of SIFIB were comparable to those of the IPACK + ACB combination. Given its technical simplicity and ability to target multiple nerves with a single injection, SIFIB may be considered a practical alternative for postoperative analgesia in TKA. These findings may assist clinicians in selecting regional anesthesia strategies when procedural limitations exist.

## 1. Background

Postoperative pain is a significant concern after total knee arthroplasty (TKA). Approximately 60% of patients experience severe pain, while 30% report moderate pain.^[1]^ Postoperative pain is intricately linked to patient satisfaction, recovery outcomes, and overall results. Multimodal analgesia is recommended to manage acute postoperative pain and reduce the risk of chronic pain.^[2,3]^

The knee joint receives innervation from both the sacral and lumbar plexus, making its nerve supply complex. The innervation of the surgical field is supplied by the femoral nerve (FN), obturator nerve (ON), sciatic nerve (SN), and lateral femoral cutaneous nerve (LFCN). In the context of postoperative analgesia, these nerves are subjected to blockade during the perioperative period to facilitate effective pain management.^[4]^

The suprainguinal fascia iliaca block (SIFIB) targets the lumbar plexus, including the femoral, lateral femoral cutaneous, and ONs. It is believed to exert its effect in a manner akin to an anterior lumbar plexus block.^[4]^ Currently, innovative techniques such as combined blocks are being advanced for the management of postoperative pain following knee arthroplasty. One method involves integrating local anesthetic injection between the popliteal artery and the posterior knee capsule (IPACK) together with adductor canal block (ACB), and in this combination, IPACK represents the periarticular local anesthetic technique.^[5]^ The ACB method involves the administration of local anesthetic around the saphenous nerve located in the adductor canal. However, when utilized in isolation, it fails to adequately relieve pain in the posterior aspect of the knee. Consequently, clinicians often incorporate the IPACK block in conjunction with the ACB to enhance pain management outcomes.^[6]^

Postoperative pain arises from tissue damage incurred during surgical procedures, leading to an inflammatory response. Surgical trauma induces cellular damage, which triggers the release of inflammatory mediators. The mediators activate nerve endings, resulting in the perception of pain. Furthermore, throughout this process, the body’s defense mechanisms are engaged to combat tissue healing and mitigate the risk of infection. The extent of inflammation can fluctuate based on the degree of surgical trauma, which may correlate directly with the intensity of pain experienced. More significant traumas can result in a heightened inflammatory response, consequently leading to increased pain severity.^[7]^

In clinical practice, biomarkers derived from hemogram parameters, including the neutrophil/lymphocyte ratio (NLR), platelet/lymphocyte ratio (PLR), and systemic immune inflammation score (SII), serve as indicators of inflammation and assist in predicting disease severity across various cases.^[8]^ Following surgical procedures, an elevation in lactate levels – which frequently serve as a marker for hypoperfusion, tissue hypoxia, or metabolic stress, may be observed. Postoperative pain can stimulate the sympathetic nervous system, causing stress responses and increasing tissue oxygen demand.^[9]^ Since the 1930s, serum C-reactive protein (CRP) levels have served as a clinical indicator of inflammation. CRP levels are elevated during inflammatory processes, including surgical interventions, infections, or traumatic events. During the postoperative phase, pain typically manifests as a component of the inflammatory response, and it is hypothesized that a correlation may exist between CRP levels and pain intensity. Elevated CRP levels may suggest heightened inflammation within the tissues and correlate with the intensity of the associated pain.^[10]^ Bupivacaine is a local anesthetic with known myotoxic effects, and serum CPK levels are used to evaluate myotoxicity.^[11]^

Although the IPACK + ACB combination is widely recommended to address both anterior and posterior innervation of the knee joint, performing 2 separate procedures may not always be practical due to time or resource constraints. In contrast, SIFIB is a single-injection, superficial block that targets components of the lumbar plexus and may offer a simpler, yet comparably effective, alternative for postoperative analgesia. Evaluating whether SIFIB can serve as a suitable substitute for the IPACK + ACB combination may assist clinicians in cases where combined techniques are not feasible or advisable.

The objective of this study was to compare the effect of the SIFIB and IPACK + ACB combination on postoperative pain scores at 24 hours following knee arthroplasty. We also investigated the effects of both regional anesthesia modalities on inflammatory markers derived from hemogram parameters, as well as other mediators and biochemical parameters associated with myotoxicity. The hypothesis we proposed was the null hypothesis.

## 2. Methods

### 2.1. Study design

The study was structured as a prospective observational investigation. The study was conducted from July 2024 to September 2024, following the approval of the Samsun University Clinical Research Ethics Committee, reference number GOKAEK 2024/10/1, and Clinical Trials registration (NCT06498557 07/06/024). The research was carried out in compliance with the principles outlined in the Declaration of Helsinki and the STROBE guidelines.

The study included patients aged 18 to 75 years who underwent unilateral primary knee replacement surgery with spinal anesthesia and were classified as ASA I to III by the American Society of Anesthesiologists. Individuals exhibiting bleeding diathesis, those undergoing anticoagulant therapy, individuals with allergies or sensitivities to local anesthetics and opioids, those allergic to paracetamol and tenoxicam, patients with infections in the proposed block area, individuals with a history of knee surgery, those utilizing gabapentinoids or corticosteroids, patients with renal or hepatic insufficiency, individuals unable to operate the patient-controlled anesthesia device, those with suspected pregnancy, pregnant or breastfeeding individuals, as well as those who declined the procedure were excluded from the study. One group was administered postoperative SIFIB, while the other group underwent a combination of preoperative IPACK and postoperative ACB.

During the postoperative period, morphine consumption as well as its side effects including nausea and vomiting, and the degree of quadriceps weakness were recorded. Using the Numeric Rating Scale (NRS) the pain levels of the patients were assessed on a scale of 0 to 10 at rest (NRS-S) and during movement, with measurements taken at the 3rd, 6th, 9th, 12th, and 24th hours post-surgery. Dynamic pain (NRS-D) was applied to all patients by an anesthesia assistant trained by a physiotherapist at specified time intervals. No active flexion or walking was performed, passive knee flexion was performed. This movement was standardized among patients and consisted of approximately 45 degrees of passive flexion of the operated knee. This angle value was determined by consensus with orthopedists to ensure tolerability by the patient or to prevent the risk of joint stress in the early postoperative period. Pain scores were recorded at maximum flexion.

The assessment of quadriceps muscle weakness involved grading the patient’s capacity to actively extend the knee on a scale from 0 to 2 (0 indicates normal strength, where the patient is able to extend the knee against both gravity and applied resistance; 1 signifies mild weakness, as the patient can extend the knee against gravity but is unable to resist applied resistance; 2 denotes severe weakness, indicating that the patient cannot extend the knee.) For statistical analysis, the presence of quadriceps weakness was deemed to be either present (2,1) or absent (0).^[12]^

The routine hemogram parameters (Sysmex XN-3000) were utilized to compare the NLR (neutrophil/lymphocyte), PLR (platelet/lymphocyte), and SII score (platelet × neutrophil/lymphocyte) ratios at 12th and 24th postoperative hours.^[13]^ At the 12th and 24th hours, measurements of CRP, lactate in venous blood gas, and CPK levels were conducted to evaluate muscle breakdown (Beckman Coulter AU5800 model chemistry analyzer, United States.) The number of patients exhibiting CPK levels that were twice the normal range for women (145 U/L) and men (171 U/L) was assessed and analyzed for comparison. The prevalence of patients exhibiting levels 5 times higher than the established threshold for both women and men, serving as an indicator of rhabdomyolysis, was assessed and analyzed for comparative purposes.^[11]^

#### 2.1.1. Patient selection and blindness

The patients were not randomized; instead, they were allocated into 2 distinct groups in a sequential manner. The blocks were administered to the patients by a single operator, and postoperative follow-ups were conducted by an anesthesiologist who was blinded to the study. The study was designed to be assessor blinded.

### 2.2. Application of spinal anesthesia and surgical procedure

Following an application of electrocardiogram, noninvasive blood pressure monitoring, and peripheral oxygen saturation monitoring in the operating room, premedication consisting of 2 milligrams (mg) of midazolam was applied. Subsequently, 15 mg of 0.5% heavy bupivacaine (Buvasin® spinal heavy, Turkey) was administered via a 25 G Quincke spinal needle from the midline at the L_3_-L_4_/L_4_-L_5_ lumbar interspace, confirming that the sensory block was achieved at the T_10_ dermatome level. Bimalleolar TKA was conducted utilizing the standard technique.

#### 2.2.1. USG guided block applications

All procedures were conducted in a designated block room following patient monitoring, adhering to aseptic protocols. The interventions utilized 0.2% bupivacaine (Buvasin®, Turkey) administered with an 80 to 100 mm block needle (Vygon Echoplex, 21 G, Ecouen, France) under ultrasound guidance (12–18 MHz, Esaote MyLabTM30 Gold Genoa, Italy), with careful observation of local anesthetic distribution. All blocks were administered by the same anesthesiologist (SCA).

#### 2.2.2. SIFIB application

SIFIB was administered within 10 minutes following the surgical procedure, utilizing a high-frequency linear transducer. During the monitoring of the patient in the postoperative observation unit, following the preparation of the inguinal region, the probe was positioned in the oblique plane, medial to the anterior superior iliac spine. Sonographic examination revealed the skin, subcutaneous tissue, sartorius muscle, internal oblique muscle, iliacus muscle, and deep circumflex artery. The needle was then advanced from caudal to cranial, positioning it within the potential space between the fascia iliaca and iliacus muscle, where 50 mL of local anesthetic was administered in a controlled manner.

#### 2.2.3. IPACK application

IPACK block was administered 30 minutes prior to the surgical procedure in the designated regional anesthesia area within the operating room. The patient underwent monitoring and was positioned in the prone orientation following the administration of 1 mg midazolam and 7.5 mg ketamine hydrochloride intravenously for the purpose of sedoanalgesia. Following adequate preparation, the popliteal fossa was examined using a low-frequency ultrasound probe. Upon visualization of the femoral condyle within the ultrasound field, the probe was advanced proximally until the femoral shaft was identified, thereby exposing the popliteal artery. The needle was subsequently advanced from the lateral direction, precisely targeting the region between the knee joint, popliteal artery, and femur, with 25 mL of local anesthetic administered under controlled conditions.

#### 2.2.4. ACB application

The procedure was conducted within 10 minutes following the operation, while the patient was situated in the postoperative observation unit. In the supine position, the femoral artery was located by positioning the linear ultrasound probe transversely in the anteromedial area of the proximal third of the thigh, ensuring abduction and slight external rotation. The block needle was subsequently positioned from lateral to medial beneath the sartorius muscle, and 20 mL of local anesthetic was administered via aspiration to the medial aspect of the artery. The nerve within the adductor canal was evaluated using sonographic techniques.

### 2.3. Perioperative pain management

In accordance with an established analgesia protocol, patients received intravenous administration of 1 g paracetamol and 20 mg tenoxicam 30 minutes prior to the conclusion of the surgical procedure. Paracetamol was subsequently administered every 8 hours, while tenoxicam was given every 12 hours during the postoperative phase. All patients received an intravenous patient-controlled analgesia device in the recovery room. Morphine was administered at a concentration of 0.3 mg/mL in a volume of 100 mL without modifying the basal infusion. The bolus dose was set at 1 mg, and the locking time was established at 15 minutes. Patients were instructed to press the bolus button if the NRS score was equal to or >4. Opioid consumption was documented at postoperative hours 3, 6, 9, 12, and 24.

### 2.4. Outcome measurements

The primary outcome was pain scores (NRS) at rest and during movement at 24 hours following surgery and secondary outcomes were the 24-hour opioid consumption, NLR, PLR, SII, CRP, lactate, and CPK values.

### 2.5. Sample size and statistical analysis

The sample size was determined using the G*Power 3.1.9.7 program. The calculations derived from a comparable study revealed a sample size of 64, with α = 0.05, power = 0.95 and effect size = 0.916.^[14]^ The study design involved the establishment of 2 equal groups, and taking into account the anticipated losses, it was determined that each group would consist of 35 individuals. The statistical analysis was conducted using IBM Statistical Package for Social Sciences (SPSS), Version 21 (IBM Corp., Armonk). The Kolmogorov–Smirnov test was employed to assess the normality of the data distribution. Descriptive statistics were presented as mean, standard deviation (Mean ± SD), median, minimum, maximum, and quartiles (25th, 75th percentile). In cases where the data exhibited a normal distribution, the *t*-test for 2 independent groups was utilized. Conversely, for data that did not adhere to normal distribution, the Mann–Whitney U test, a non-parametric method, was employed. The Chi-square test was employed to conduct a comparison between the groups. *P* < .05 was deemed statistically significant.

## 3. Results

**Figure 1.**
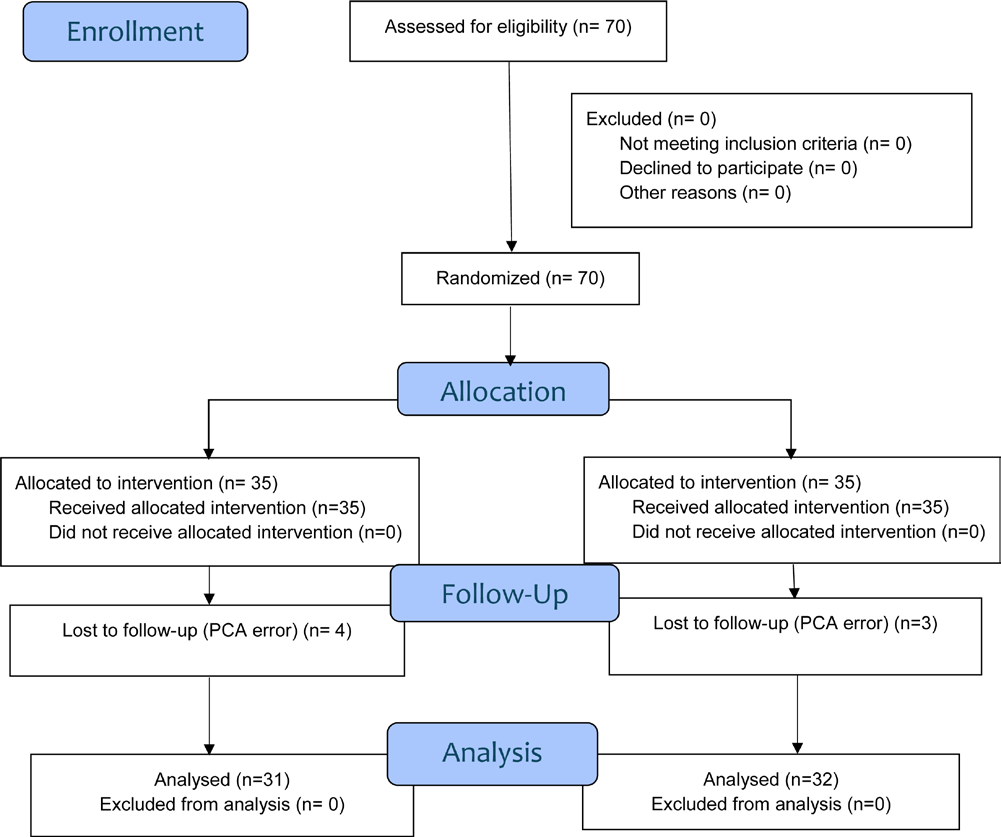
Consort flow chart. ACB = adductor canal block, IPACK = local anesthetic infiltration between the popliteal artery and the posterior capsule of the knee, n = number, SIFIB = suprainguinal fascia iliaca block.

Seventy patients undergoing primary TKA were included in the study. During the postoperative follow-up period, 4 patients in the SIFIB group and 3 patients in the IPACK + ACB group had patient-controlled analgesia problems, so these patients were excluded from the study. As a result, data from 31 patients in the SIFIB group and 32 patients in the IPACK + ACB group were analyzed (Fig. 1). There were no differences between the groups in terms of patient age, gender, ASA classification, body mass index, and surgery duration (Table 1).

**Table 1:** Demographic data.

	SIFIB (n = 31)	IPACK + ACB (n = 32)	*P*
Age (yr)	64.52 ± 6.63	66.16 ± 6.04	.18
Gender (male/female) (n)	4/27	6/26	.732
ASA (I/II/III) (n)	3/26/2	3/22/7	.196
Height (cm)	162.71 ± 8.39	162.6 ± 8.85	.805
Weight (kg)	86.26 ± 9.77	91.94 ± 10.11	.22
BMI (kg/m^2^)	32.82 ± 5.01	34.79 ± 4.99	.124
Surgical time (min)	50.48 ± 3.95	51.09 ± 4.88	.72

ACB = adductor canal block, ASA = American Society of Anesthesiology, BMI = body mass index, IPACK = local anesthetic infiltration between the popliteal artery and the posterior capsule of the knee, n = number, SIFIB = suprainguinal fascia iliaca block.

### 3.1. Pain scores

Pain scores at rest (NRS-S) and during movement (NRS-D) were recorded at the 3rd, 6th, 9th, 12th, and 24th postoperative hours. There were no statistically significant differences between the groups in either NRS-S or NRS-D at any of these time points. This indicates that the 2 regional anesthesia techniques provided comparable levels of analgesia throughout the early postoperative period (Table 2).

**Table 2:** Comparison of pain scores at rest (NRS-S) and on movement (NRS-D) for each time period of patients in the SIFIB and IPCK + ACB groups.

NRS-S	SIFIB (n = 31)	IPACK + ACB (n = 32)	*P*
3rd hour	3 (1–4)	3 (1.25–5)	.76
6th hour	3 (2–6)	4 (2–5)	.79
9th hour	3 (1–4)	3 (2–5)	.31
12th hour	2 (1–3)	2.5 (1.25–3)	.21
24th hour	2 (1–3)	2 (1–3)	.69
NRS-D			
3rd hour	4 (2–5)	4 (2–6)	.44
6th hour	4 (2–6)	5 (3–6)	.40
9th hour	3 (2–4)	4 (3–5.75)	.15
12th hour	3 (2–4)	4 (3–4)	.51
24th hour	3 (2–4)	3 (2–4)	.32

Data are expressed as median (25–75 percentile).

ACB = adductor canal block, IPACK = local anesthetic infiltration between the popliteal artery and the posterior capsule of the knee, n = number, NRS = Numeric Rating Scale, SIFIB = suprainguinal fascia iliaca block.

### 3.2. Opioid consumption

**Figure 2.**
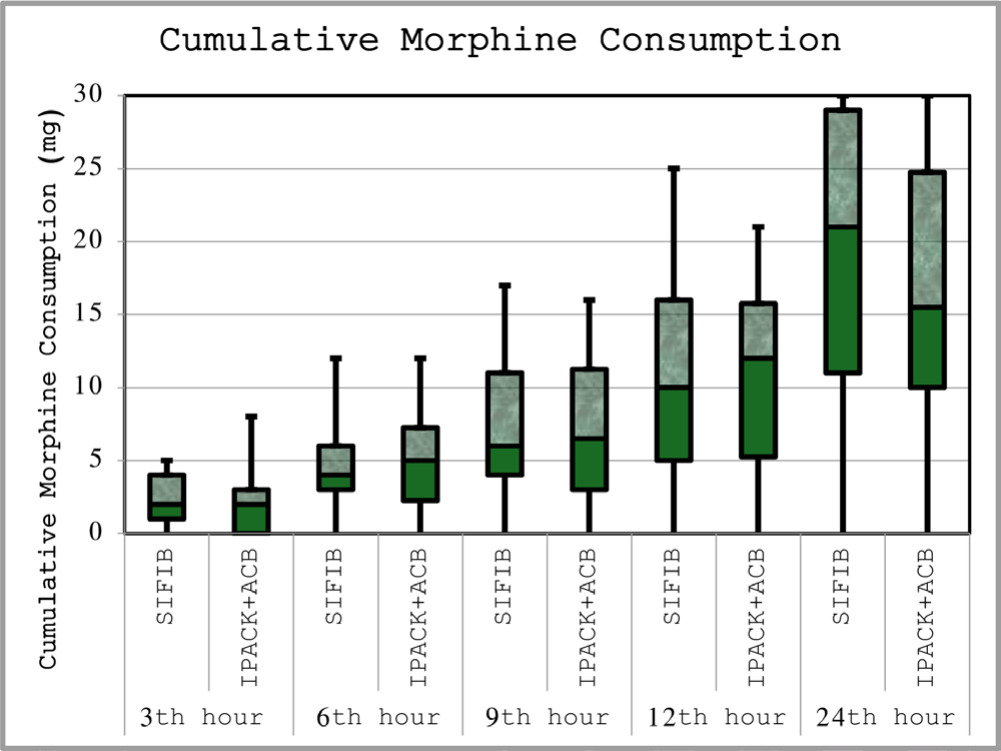
Box plot comparison of cumulative morphine consumption between groups at different time periods. ACB = adductor canal block, IPACK = local anesthetic infiltration between the popliteal artery and the posterior capsule of the knee, n = number, SIFIB = suprainguinal fascia iliaca block.

The total morphine consumption for patients over a 24-hour period was similar between SIFIB and IPACK ± ACB groups (19 ± 8.73 mg vs16.78 ± 8.04 mg, *P* = .397). The evaluation of cumulative morphine consumption among the patients indicated that the levels were consistent across all time periods (Fig. 2). The cumulative consumption of morphine was analyzed for the 0 to 12 hour and 12 to 24 hour intervals across each group, with no differences noted (Table 3).

**Table 3:** Comparison of cumulative morphine consumption of patients at 0–12 and 12–24 h between groups.

	SIFIB	IPACK + ACB	*P*
0–12 h	10.77 ± 6.71 (mg)	10.25 ± 5.79 (mg)	.78
12–24 h	8.23 ± 4.65 (mg)	6.84 ± 4.62 (mg)	.18

Data are demonstrated as mean ± standard deviation.

ACB = adductor canal block, IPACK = local anesthetic infiltration between the popliteal artery and the posterior capsule of the knee, SIFIB = suprainguinal fascia iliaca block.

### 3.3. Inflammatory markers

**Figure 3.**
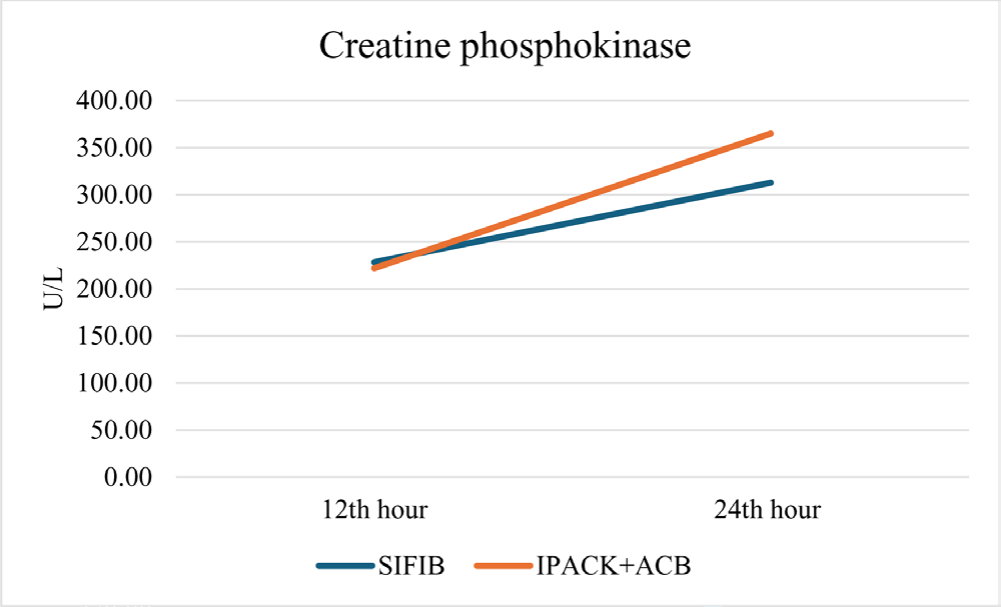
Mean creatine phosphokinase levels of the groups. ACB = adductor canal block, CPK = creatine phosphokinase, IPACK: Local anesthetic infiltration between the popliteal artery and the posterior capsule of the knee, n = number, SIFIB = suprainguinal fascia iliaca block, U = unit.

Inflammatory biomarkers derived from blood samples collected from patients were analyzed at the 12th and 24th hours. The comparison of NLR, PLR, SII, CRP, Lactate, and CPK levels between the 2 groups revealed no significant differences (Table 4). To assess muscle damage, the count of patients exhibiting CPK levels exceeding twice the reference value for each group (calculated separately for women and men) was established and analyzed for comparison. In a similar manner, patients exhibiting CPK levels exceeding 5 times the reference value, indicative of rhabdomyolysis, were identified, and the patient counts were compared across the groups. No significant difference was observed between the groups regarding the number of patients with CPK levels exceeding 2 times and 5 times the normal range. Specifically, the SIFIB group had 5 patients with CPK values 5 times higher, while the IPACK + ACB group had 9 patients. This difference did not reach statistical significance (*P* = .453). Figure 3 illustrates the graph depicting the increase in CPK levels (Fig. 3).

**Table 4:** Comparison of laboratory parameters of patients at 12 and 24 h between groups.

12th hour	SIFIB (n = 31)	IPACK + ACB (n = 32)	*P*
NLR	5.9 ± 2.63	7.76 ± 9.04	.54
PLR	178.4 ± 79.33	180.5 ± 57.62	.48
SII	1330.67 ± 672.83	2156.13 ± 4964.4	.79
CRP (mg/ L)	37.8 ± 35.99	37.99 ± 29.6	.69
Lactate (mmol/L)	1.5 ± 0.56	1.74 ± 0.53	.92
CPK (U/ L)	228.33 ± 203.82	221.92 ± 263.33	.51
24th hour			
NLR	6.02 ± 3.52	6.55 ± 3.12	.35
PLR	179.4 ± 115.25	191.5 ± 80.14	.195
SII	1414.77 ± 1464.7	1418.09 ± 871.38	.398
CRP (mg/L)	117.35 ± 78.47	93.29 ± 39.94	.257
Lactate (mmol/L)	1.74 ± 0.49	1.7 ± 0.41	.685
CPK (U/ L)	312.77 ± 293.32	364.92 ± 429.1	.864

Data are presented as mean ± standard deviation.

ACB = adductor canal block, CPK = creatine phosphokinase, CRP = C-reactive protein, IPACK = local anesthetic infiltration between the popliteal artery and the posterior capsule of the knee, n = number, NLR = neutrophil/lymphocyte ratio, PLR = platelet/lymphocyte ratio, SIFIB = suprainguinal fascia iliaca block, SII = systemic immune inflammation score, U = unit.

### 3.4. Quadriceps weakness

Upon evaluation of quadriceps weakness at the 24-hour mark, it was noted that 17 patients in the SIFIB group and 21 patients in the IPACK + ACB group exhibited no signs of weakness. Conversely, weakness was identified in 14 patients from the SIFIB group and 11 patients from the IPACK + ACB group. The analysis revealed no significant difference between the groups regarding quadriceps weakness (*P* = .537) (Table 5).

**Table 5:** Comparison of quadriceps weakness of patients at 24 h.

Group		SIFIB (n = 31)	IPACK + ACB (n = 32)	Total (n = 63)
Quadriceps weakness	0	17	21	38
	1 – 2	14	11	25

Chi square = 0.381, *P* = .537.

ACB = adductor canal block, IPACK = local anesthetic infiltration between the popliteal artery and the posterior capsule of the knee, n = number, SIFIB = suprainguinal fascia iliaca block.

## 4. Discussion

Our study comparing the efficacy of SIFIB and IPACK + ACB for postoperative analgesia in TKA revealed that the pain scores of patients, both at rest and during movement, were comparable across each hour of assessment. No significant differences were noted between the groups regarding the 24-hour morphine consumption of the patients. The inflammatory markers NLR, PLR, SII, CRP, and lactate values obtained from blood samples collected from patients at the 12th and 24th hours postoperatively exhibited comparable results. The comparison of CPK values, assessed as an indicator of muscle breakdown, revealed no significant differences between the groups. Upon further examination of CPK levels to assess rhabdomyolysis, we observed no significant difference between the groups regarding the number of patients exhibiting CPK values that exceeded 5 times the normal limit at the 12th and 24th hours. The strength levels of the quadriceps, assessed as a measure of motor loss following the block, were comparable in both groups.

Multimodal analgesia is advised in enhanced recovery after surgery (ERAS) protocols for patients undergoing total hip arthroplasty (THA) to ensure effective pain management and, consequently, improved patient outcomes with expedited rehabilitation.^[15]^ Inadequate management of postoperative pain following TKA surgery can lead to delayed recovery and diminished patient satisfaction. Nerve block techniques, including the IPACK block and ACB, have demonstrated efficacy in providing analgesia for TKA. Guo et al^[6]^ demonstrated that patients administered a combination of IPACK and ACB experienced significantly lower pain scores within the first 24 hours post-surgery in comparison to those who underwent general anesthesia alone. Albrecht et al^[1]^ demonstrated in their meta-analysis that there is moderate evidence supporting the efficacy of IPACK in providing analgesia for the posterior aspect of the knee 12 hours post-TKA; however, no other clinically significant benefits were identified in terms of analgesic or functional outcomes.^[1]^ Another study demonstrated that the IPACK block effectively reduces opioid consumption as a complementary intervention following THA. Additionally, it was found that both the ACB and the IPACK block yield superior physical therapy outcomes compared to the FN block (FNB) alone, as well as the combination of FNB and IPACK block, and facilitate earlier hospital discharge.^[16]^ Chan et al^[17]^ concluded that the IPACK block serves as a complementary technique to the ACB, offering a motor-sparing regional anesthesia option for knee prosthesis surgery. Given that ACB alone does not adequately provide analgesia, particularly for the posterior region of the knee, it is advisable to combine it with techniques such as IPACK or LIA.^[18,19]^

In this study, we found that the perioperative analgesic efficacy and safety of SIFIB were similar to the efficacy and safety of the ACB + IPACK combination in knee surgery. This finding may have an anatomical basis. It is known that when SIFIB is performed with high volumes, the ON is also blocked. The deep branch of the ON extends to the popliteal fossa together with the articular branches of the sciatic nerve, between the popliteal artery and the posterior aspect of the knee capsule, and contributes to the popliteal plexus, which is the target of the IPACK block.^[6]^ Therefore, when we apply SIFIB, we not only block the anterior compartments of the knee, but also partially block the posterior innervation of the joint capsule. This anatomical explanation may support the logical consistency of our results. Additionally, SIFIB may be considered an alternative to the ACB + IPACK combination due to advantages such as requiring only a single injection and being a relatively superficial technique. Previously, a retrospective study conducted in our clinic showed that adding an IPACK block to SIFIB did not provide any additional clinical benefit.^[12]^ With this current study, we aimed to demonstrate that SIFIB alone may be a viable alternative to commonly recommended combinations. In practice, anesthesiologists and surgeons often hesitate to perform an IPACK block at the end of surgery due to its proximity to the surgical field. As a result, part of the block’s analgesic effect may occur under general or spinal anesthesia, limiting its clinical usefulness. This concern may be avoided with the use of SIFIB.

Based on the findings from these studies, we implemented the combination of IPACK and ACB in one group and SIFIB in the other group of patients. Research conducted by Kefeli Çelik et al^[4]^ demonstrates that SIFIB serves as an effective analgesic technique in knee replacement surgery. The findings indicate that SIFIB significantly decreased 24-hour morphine consumption and contributed to a more comfortable postoperative experience.

Our study indicates that SIFIB versus combination of IPACK and ACB yielded comparable NRS scores at rest and during movement following TKA surgery. Additionally, morphine consumption among patients in both groups was found to be similar. Vermeylen et al^[20]^ indicated that a volume of 40 mL of local anesthetic was necessary to effectively block the targeted nerves (FN, LFCN, and ON) in their study, which involved magnetic resonance imaging and sensory block analysis conducted on volunteers. In a separate cadaver study, the minimum effective volume (MEV 90) necessary to achieve complete blockade of the FN, LFCN, and ON during the application of SIFIB was documented as 62.5 mL.^[21]^ This study demonstrated that SIFIB with 50 mL of 0.20% bupivacaine delivered analgesia that was as effective as the combination of IPACK and ACB, which is recommended as a standard in the multimodal analgesia protocol for postoperative analgesia in THA surgery.

Numerous studies have established a correlation between serum inflammatory mediators, cytokines, acute phase reactants, stress metabolites, and muscle damage markers with the occurrence of postoperative acute pain, which is a response to inflammation induced by surgical procedures. The presence of inflammatory cytokines, inflammatory biomarkers, muscle damage markers, and stress metabolites may influence the formulation of the postoperative analgesic regimen.^[8,22,23]^ The markers are utilized in the assessment of various postoperative analgesia techniques and the subsequent outcomes following surgery. In this study, we evaluated the relationship between NLR, PLR, SII, CRP, and lactate levels and postoperative pain. No significant differences were observed in these markers between the SIFIB group and the IPACK + ACB group. These findings were consistent with the absence of significant variation in NRS scores and morphine consumption.

Bupivacaine, similar to other local anesthetics, presents a potential risk of myotoxicity. Myotoxicity has the potential to induce mitochondrial dysfunction within muscle cells, leading to elevated intracellular calcium levels and subsequent necrosis of these cells. The damage is typically reversible; however, it may result in permanent damage when subjected to high doses or repeated applications. The observed effects may be attributed to the lipophilic properties and prolonged duration of action of bupivacaine, which are typically associated with the direct injection of local anesthetics into muscle tissue.^[24,25]^ In SIFIB, the local anesthetic is administered to the fascial plane rather than directly to the muscle; nonetheless, absorption from the fascial area into the muscle will occur. In ACB and IPACK blocks, the proximity of the local anesthetic to the muscle may result in increased myotoxicity. In our study, we observed that the number of patients exhibiting CPK levels 5 times higher than normal, which are indicative of rhabdomyolysis, was 5 in the SIFIB group and 9 in the IPACK + ACB group; however, this difference did not reach statistical significance. In a study by Unal et al,^[11]^ an elevation in CPK levels was noted in patients who underwent SIFIB; however, no statistically significant difference was found when compared to the control group that did not receive the block. Rakhi et al^[24]^ assessed the myotoxicity risk associated with local anesthetics, comparing patients who received 2 distinct fascial plane blocks and those who did not. The findings revealed significantly elevated CPK levels in patients who underwent the block procedure when compared to baseline values. In our investigation, the anatomical region where the block was administered exhibited no significant differences regarding myotoxicity.

The quadriceps muscle is a significant muscle that is innervated by the FN and is responsible for the extension of the knee joint. The blockade of the FN during SIFIB may result in a temporary reduction in muscle strength. While research has shown that the likelihood of nerve involvement is minimal, it is possible to observe quadriceps muscle weakness in certain patients. The side effect is typically transient, with muscle function returning to its normal state once the effects of the blockage have subsided. The utilization of USG by skilled practitioners is crucial to mitigate this side effect.^[25,26]^ ACB is conducted by temporarily obstructing the saphenous nerve, which is one of the terminal branches of the FN. An essential benefit of ACB is its efficacy in managing pain around the knee while minimizing the risk of inducing weakness in the quadriceps muscle, as it selectively targets the sensory branch of the FN, leaving the motor fibers unaffected. Research indicates that quadriceps muscle weakness is minimal with the use of ACB, suggesting that this block presents a safer alternative to FNBs. This block is particularly favored for patients aiming to mobilize early and maintain muscle strength.^[27,28]^ The IPACK block specifically targets the genicular nerve branches located in the popliteal fossa, which play a crucial role in pain transmission at the posterior knee. This intervention effectively alleviates pain while preserving muscle function.^[16,29]^ While the literature suggests a potential for increased quadriceps weakness in the SIFIB group, our study revealed comparable instances of weakness across both groups. Interestingly, in contrast to existing findings, a greater number of patients in the SIFIB group exhibited no muscle weakness at all.

In our study, the absence of a control group and the lack of randomization may present limitations; however, we implemented a consecutive patient selection process, ensuring that our patients exhibited comparable demographic characteristics. One additional limitation is that the patients were not blinded to the applied blocks; however, we endeavored to mitigate bias by blinding the evaluator. The subjective nature of pain scoring, particularly without full patient blinding, may have introduced bias. In addition, although passive knee flexion was applied for dynamic pain assessment, the lack of a fully standardized movement protocol may have affected the reproducibility of NRS-D scores. The relatively short follow-up period is another limitation, as longer-term differences between groups may not have been captured. Regarding secondary outcomes, while CPK levels were used as a marker of myotoxicity, the clinical significance of these findings remains uncertain. Our study was not powered to detect differences in secondary biochemical outcomes, and no statistically significant differences were observed. Moreover, although the study could have been strengthened by including glutamate levels alongside CPK, this was not possible due to financial limitations. Finally, while we aimed to assess quadriceps strength more objectively, we were unable to use a handheld dynamometer due to equipment unavailability.

## 5. Conclusion

Our findings indicate that the combination of ACB + IPACK and SIFIB alone provided comparable postoperative analgesic effects in patients undergoing TKA. Both techniques also resulted in similar outcomes with respect to inflammatory markers, myotoxicity (as reflected by CPK levels), and quadriceps strength. These results suggest that SIFIB, despite being a single-injection technique, may serve as a practical alternative to the more commonly used ACB + IPACK combination, particularly in clinical settings where procedural simplicity, limited time, or restricted access to resources make multiple block applications less feasible. In addition, the anatomical spread achieved with high-volume SIFIB may partially involve posterior innervation through the ON’s deep branch, potentially explaining its similar analgesic effect. Future randomized controlled studies with larger sample sizes and longer follow-up periods are warranted to validate these findings and better define the clinical role of SIFIB in comparison to combined block techniques.

## Acknowledgments

The authors declare that they used ChatGPT (OpenAI, San Francisco, CA, USA) to assist with language editing and clarity improvements during the manuscript revision process.

## Author contributions

**Conceptualization:** Şenay Canikli Adigüzel, Caner Genç, Ebru Kayikçi, Ahmet S. Genç, Serhat Durusoy, Adem Yildiz, Serkan Tulgar.

**Data curation:** Şenay Canikli Adigüzel, Caner Genç.

**Formal analysis:** Şenay Canikli Adigüzel.

**Investigation:** Şenay Canikli Adigüzel.

**Methodology:** Şenay Canikli Adigüzel, Ebru Kayikçi, Serkan Tulgar.

**Project administration:** Şenay Canikli Adigüzel.

**Writing – original draft:** Şenay Canikli Adigüzel.

**Writing – review & editing:** Serkan Tulgar.
